# Recent Advances in Chiral Gold Nanomaterials: From Synthesis to Applications

**DOI:** 10.3390/molecules30040829

**Published:** 2025-02-11

**Authors:** Huangsiyu Chen, Changlong Hao

**Affiliations:** School of Food Science and Technology, State Key Laboratory of Food Science and Resources, Jiangnan University, Wuxi 214122, China

**Keywords:** chiral, gold nanomaterials, synthesis, sensor, catalysis, nanomedicine

## Abstract

In recent years, the field of chiral gold nanomaterials has witnessed significant advancements driven by their unique properties and diverse applications in various scientific domains. This review provides an in-depth examination of the synthesis methodologies and evolving applications of chiral gold nanomaterials, which have emerged as vital tools in areas such as antibacterial therapies, biosensing, catalysis, and nanomedicine. We start by discussing various synthesis techniques, focused on seed-mediated growth and circularly polarized light-assisted methods, each contributing to the controlled synthesis of chiral gold nanostructures with tailored optical activities. This review further delves into the applications of these nanomaterials, showcasing their potential in combating antibiotic-resistant bacteria, improving cancer immunotherapy, promoting tissue regeneration, and enabling precise biosensing through enhanced sensitivity and selectivity. We highlight the fundamental principles of chirality and its critical role in biological systems, emphasizing the importance of chiral gold nanomaterials in enhancing optical signals and facilitating molecular interactions. By consolidating recent findings and methodologies, this review endeavors to illuminate the promising future of chiral gold nanomaterials and their critical role in addressing contemporary scientific challenges.

## 1. Introduction

Chirality, as a fundamental property describing the asymmetry of objects, is crucial across multiple scientific disciplines including chemistry, biology, and materials science. Biological macromolecules such as proteins, nucleic acids, and amino acids are almost exclusively chiral, typically existing in a single configuration to ensure their specific biological activity. For example, amino acids predominantly occur in the L-configuration, while sugars are found in the D-configuration in nature. This chirality selection not only influences protein folding and function but also affects enzyme catalysis reactions and the binding between receptors and ligands. Hence, investigating chirality holds substantial importance in exploring biological activities and therapeutic interventions for diseases. In recent decades, considerable advancements have been achieved in the study of inorganic nanoparticles that exhibit stronger chiral signals in the visible and near-infrared regions.

Within the realm of nanoscale materials, chirality has garnered significant interest because of its influence on optical, electronic, and catalytic behaviors [1,2,3]. Chiral gold nanomaterials have received particular attention from researchers due to their unique surface plasmon resonance characteristics, excellent biocompatibility, and tunable chiral activity. Chiral gold nanomaterials possess remarkable surface plasmon resonance features [4], and by altering the shape and size of these particles, it is possible to modulate their surface plasmon resonance properties, making them an ideal choice for developing novel optical sensors and biomarkers. The development of synthesis methods for chiral gold nanomaterials has played a key role in our understanding of chirality within these materials. With the advent of more advanced approaches, such as seed-mediated growth [5] and circularly polarized light (CPL)-assisted synthesis [6], significant advancements have been achieved. These innovations not only enable the production of chiral gold nanomaterials with improved uniformity and optical activity but also pave the way for exploring new applications driven by chirality, such as catalysis, nanomedicine, and biosensing.

In recent years, researchers have synthesized a large number of chiral gold nanomaterials using chiral ligands as chiral inducers through various wet chemical methods, which have been applied in asymmetric catalysis, chiral sensing, enantiomer separation, and tumor therapy, among other areas. This review focuses on the construction of structurally chiral gold nanomaterials over the past five years and their latest developments in related fields.

## 2. Synthesis of Chiral Gold Nanomaterials

The preparation of chiral gold nanomaterials has garnered widespread attention due to their significant implications in a wide range of potential applications, ranging from biomedical fields to enantioselective photocatalytic reactions [7,8,9]. A plethora of chiral gold nanostructures with unique optical properties have been prepared, with their dimensions, shapes, and morphologies meticulously controlled [5,10,11,12,13,14,15]. To fabricate chiral gold nanostructures, various manufacturing strategies have been devised, primarily including (1) template-based methods, which involve employing template molecules or supramolecular structures to guide the self-assembly and alignment of nanomaterials. For instance, DNA and chiral small molecules serve as templates, co-assembling with gold nanoparticles to form chiral gold nanoassemblies [16,17,18,19,20,21,22,23]. For instance, Lu et al. organized gold nanorods in a long-range manner similar to liquid crystals, specifically within helical assemblies combined with human islet amyloid polypeptides [24]. The improvement in long-range order provides valuable structural guidance for designing materials with high optical asymmetry, opening new avenues for biochemical applications. (2) Circularly polarized light (CPL)-driven synthesis involves irradiating chloroauric acid solutions with left-handed or right-handed circularly polarized (LCP or RCP) light to synthesize chiral gold nanoparticles [25]. (3) Seed-mediated growth method involves selecting gold nanoparticles of appropriate size as seeds and incorporating suitable chiral molecules (including amino acids, peptides, proteins, DNA, etc.) into the growth solution to participate in the reaction, which can endow the final product with chiral characteristics. By altering parameters in the reaction environment, such as temperature, pH, solvent, and molar ratios, precise control over the size and structure of chiral Au nanomaterials can be achieved.

These diverse chiral construction methods have been extensively studied, each with its own merits and demerits. While template-based methods represent a significant approach for fabricating chiral gold nanostructures, they have been comprehensively reviewed elsewhere. In the next part, we will concentrate on the synthesis of structurally chiral, monodisperse gold nanomaterials.

### 2.1. Seed-Mediated Growth Method

The seed-mediated growth method offers a facile approach for producing chiral gold nanomaterials with enhanced chiroptical properties [26,27,28,29,30]. A variety of chiral gold nanomaterials have been synthesized by selecting different starting nanomaterials as seeds, such as a gold nanorod [31,32,33,34,35,36], gold nanodisk [37], gold nanocube [38], gold octapod [39], gold rhombicuboctahedron [40,41], or gold decahedra [42]. The introduction of chiral ligands, such as amino acids, DNA, and peptides, into the seed solution system plays a crucial role in modulating the optical activity of chiral gold nanomaterials, as they not only provide a source of chirality but also enhance coupling strength through amplified electromagnetic fields. This is achieved by positioning chiral molecules in hotspots (nanogaps or nanocavities), initiating the twisted surface formation of gold nanomaterials through helical structural effects.

A common synthetic strategy involves using gold nanoparticles with exposed low-index facets as seeds, followed by the addition of chiral ligands (mainly thiol-containing molecules such as cysteine, penicillamine, and glutathione, as well as non-thiol chiral ligands such as alkaloids) to the growth solution. As gold ions (Au^3+^) are reduced, the chiral ligands interact with high-index surfaces to control the chiral shape of gold nanoparticles, with gold nanoparticles exposing low-index facets growing into chiral gold nanomaterials exposing high-index facets. The composition of the growth solution (such as peptide sequences and concentrations) and the morphology of the seed nanoparticles influence the growth kinetics, with gold–sulfur bonds and other functional groups in amino acids or peptides participating in the nanoparticle growth process, inducing the dynamic morphological evolution of gold seed nanoparticles from low-index plane exposure to chiral nanoparticles.

**Figure 1:**
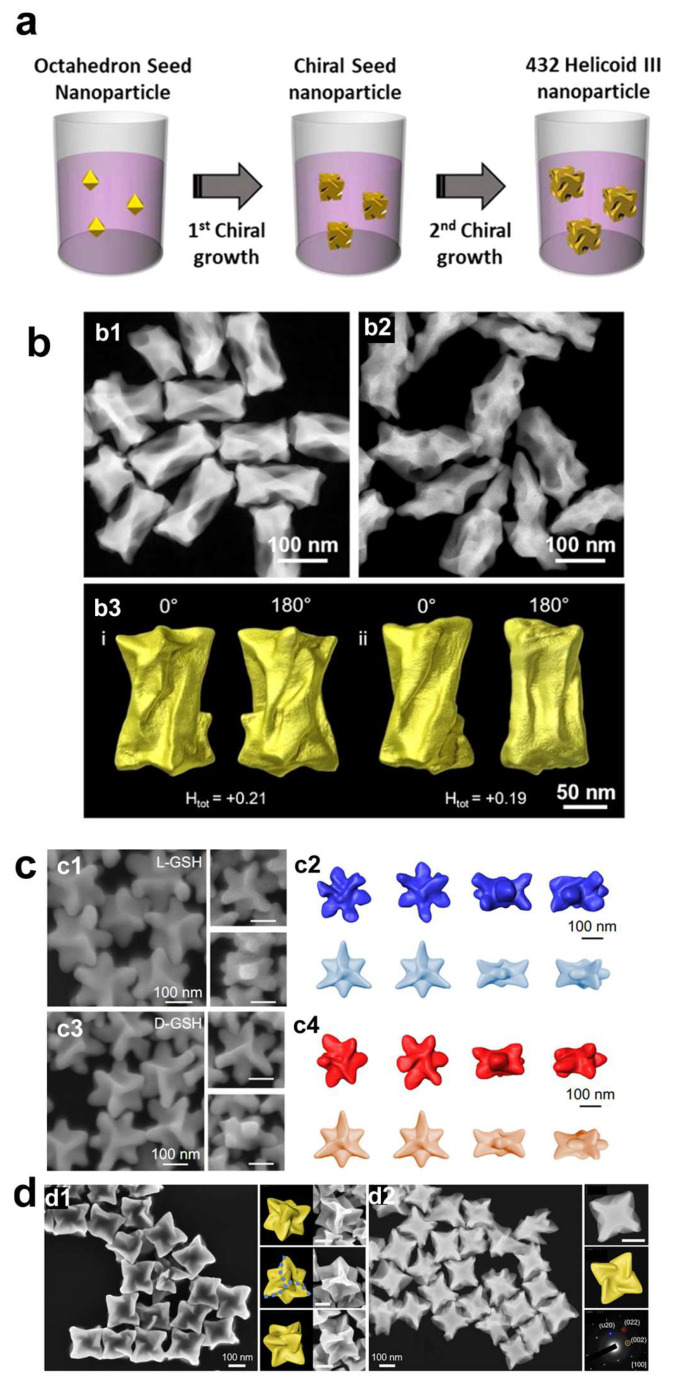
(**a**) Synthetic steps of devised multi-chiralityevolution step synthesis of 432 helicoid III [10]. (**b**) Characterization of chiral Au NRs [35]. Representative high angle annular dark field scanning transmission electron microscopy (HAADF STEM) images of chiral (**b1**) SC-Au NRs and (**b2**) PT-Au NRs. (**b3**) Isosurface renderings of electron tomography reconstructions for typical chiral SCAu NRs. (**c**) Shape of chiral Au nanotriskelions [37]. (**c1**) SEM images of the Au-L-nanotriskelions. (**c2**) Tomography reconstruction (**top**) and constructed model (**bottom**) of the Au-L-nanotriskelions. (**c3**) SEM images of the Au-D-nanotriskelions. (**c4**) Tomography reconstruction (**top**) and constructed model (**bottom**) of the D-nanotriskelions. (**d**) Shape of the chiral concave vortex cube-shaped Au nanostructures (VC-I) [40]. (**d1**) SEM images of chiral Au VC-I. (**d2**) HAADF-STEM images, geometric model, and the corresponding SAED pattern of a single chiral VC-I. Scale bars: 100 nm.

The shape of the intermediate seed plays a crucial role in the development of chiral structures [7,8,9]. Gold octahedrons and nanocubes are two common seed materials [5,10,11,12,14,15]. Lee et al. introduce a novel method for the preparation of chiral Au nanomaterials, utilizing amino acids and peptides to regulate their chirality [5]. The authors employ a two-step aqueous-based growth method that utilizes organothiol additives, specifically cysteine and cysteine-based peptides, to achieve high-Miller-index surfaces that are inherently chiral. The resulting gold nanoparticles exhibit intense chiral plasmonic optical activity, with a dissymmetry factor of approximately 0.2. The dissymmetry factor, or g-factor, is defined as the ratio between the difference and the sum of the intensities of left- and right-handed circularly polarized light absorbed by a chiral molecule or material. This factor is crucial for quantifying the optical asymmetry of chiral materials, such as the plasmonic nanoparticles discussed herein. In 2020, the same group reported that by applying precisely controlled kinetic conditions to two distinct growth stages, the modified chiral gold nanoparticles exhibited significantly enhanced uniformity, achieving a higher dissymmetry factor of g = 0.31(Figure 1a) [10].

The differential growth rates of chiral high-index planes, driven by the specific interactions of the amino acids with the gold surface, lead to the formation of distinctive morphologies, including cube-like structures with twisted edges. Using the gold octahedral seed, the same group also prepared chiral gold nanoparticles using the dipeptides Glu-Cys and Cys-Gly [12]. The chiral Au nanoparticles guided by Glu-Cys have a cubic form adorned with an extending chiral wing, whereas those crafted with Cys-Gly present a shape reminiscent of a rhombic dodecahedron, featuring curved edges and elliptical indentations on each facet.

Other than peptides, single-stranded oligonucleotides can also be used as chiral shape modifiers [15]. Specifically, oligonucleotides composed of adenine nucleobases were found to induce distinct chirality in gold nanoparticles, with a dissymmetric factor of up to g ~ 0.04 in the visible wavelength range, while other nucleobases did not show such chiral development [11]. The fabricated chiral Au nanostructures demonstrated a counter-clockwise spiral of chiral arms, with each arm measuring approximately 200 nm in length.

Gold nanorods (AuNRs) are also a highly utilized starting seed. González-Rubio et al. investigate the surfactant-assisted seeded growth of anisotropic gold nanocrystals, emphasizing the role of chiral micelles formed with dissymmetric cosurfactants [31]. The mixed micelles can effectively adsorb onto gold nanorods, leading to the formation of quasihelical patterns that guide the seeded growth process. The research highlights the emergence of sharp chiral wrinkles on the nanocrystals, which are responsible for generating chiral plasmon modes characterized by a high dissymmetry factor (~0.20). Furthermore, the micelle-directed growth mechanism is demonstrated to be versatile, extending beyond gold nanocrystals to the seeded growth of chiral platinum shells on gold nanorods. This innovative approach presents a reproducible, straightforward, and scalable method for the synthesis of nanoparticles with high chiral optical activity. More recently, the same group reported that the handedness of chiral gold nanocrystals can be reversed by employing gold nanorod seeds with either single-crystalline or pentatwinned structures, even when using the same enantiomer of the chiral inducer (Figure 1b) [35]. Ni et al. reported the synthesis of chiral Au nanoparticles with a square cross-section, offering a unique perspective on growth mechanisms based on the chirality of specific facets.

In addition to utilizing various morphologies of gold nanoparticles as initial seeds, the synthesis of chiral gold nanomaterials with distinct morphologies can also be achieved through the use of halogen ions and divalent metal ions. Zheng et al. developed an innovative halide-assisted differential growth approach that promotes the asymmetric expansion of chiral gold nanoparticles possessing threefold rotational symmetry (Figure 1c) [37]. The anisotropic gold nanodisks are utilized as seed particles to produce triskelion-shaped chiral nanoparticles with tunable sizes and diverse morphologies. The research highlights the ability to manipulate the directional growth rates of the nanoparticles to create a variety of chiral morphologies when homochiral ligands are present. This versatility in design allows for the customization of the nanoparticles’ optical properties and shapes. Wan et al. introduce a novel Cu^2+^-dominated chiral growth strategy that effectively directs the formation of concave chiral gold nanoparticles (Figure 1d) [40]. This methodology underscores the pivotal function of trace Cu^2+^ ions as catalysts in the seed-mediated growth process, enabling the transference of chirality from chiral molecules to the synthesized nanoparticles. The Cu^2+^ ions selectively enhance the deposition of gold atoms along the [110] and [111] crystallographic orientations, which results in the formation of concave V-shaped structures. These discoveries have broadened our comprehension of biomolecule-guided synthesis of chiral gold nanoparticles and offered valuable perspectives on the precise synthesis of chiral nanoparticles, essential for the advancement of sophisticated optical materials.

### 2.2. Circularly Polarized Light-Driven Synthesis of Chiral Au Nanomaterials

**Figure 2:**
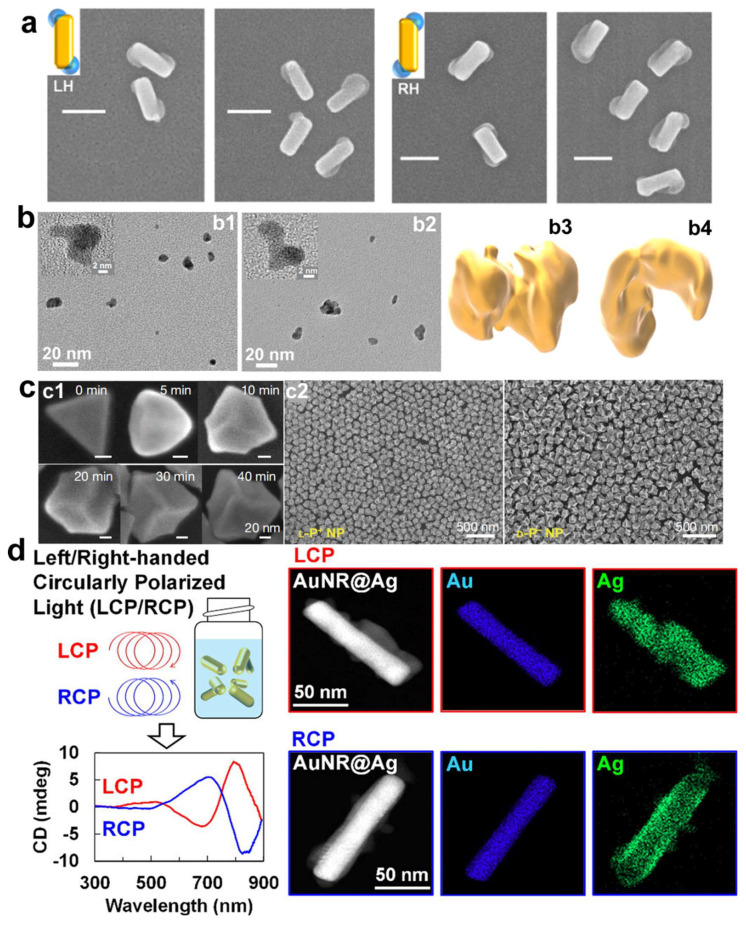
(**a**) SEM images and tomography reconstruction of chiral Au nanostructures prepared by RCP and LCP light irradiation. Scale bars: 100 nm [43]. (**b**) High-resolution TEM images and tomographic reconstructions of chiral AuNPs obtained with LCP (**b1**,**b3**) and RCP (**b2**,**b4**) illumination, respectively [44]. (**c**) Scanning electron microscope (SEM) images (**c1**) of L-P^+^ NPs (under LCP illumination) after 0, 5, 10, 20, 30, and 40 min of illumination at 594 nm with 84 mW/cm^2^. (**c2**) SEM images of L-P^+^ NPs and D-P^−^ (under RCP illumination) [25]. (**d**) Schematic illustration of the photochemical Ag growth on colloidal AuNRs (AuNRs@Ag) driven by CPL irradiation, and the CD spectra of chiral AuNRs@Ag nanomaterials and the corresponding EDS mapping images [47].

Chiral small molecules aside, chiral physical forces such as chiral light fields and magnetic fields can also be employed as chirality-inducing factors in the synthesis of chiral gold nanoparticles, with circularly polarized light (CPL) being a quintessential chiral physical force utilized in their induced synthesis [6,25,43,44,45,46,47]. In 2018, Saito and Tatsuma pioneered the use of CPL as the sole chirality-inducing factor to construct chiral plasmonic nanoparticles (Figure 2a) [43]. Non-chiral gold nano-rectangles placed on a TiO_2_ film served as precursors; upon irradiation with CPL, localized electric field distributions were generated at specific corners of the rectangles, dependent on the chirality of the CPL. Under the influence of the localized electric field, plasmon-induced charge separation occurred at particular angles of the rectangles, triggering redox reactions and leading to the deposition and growth of PbO_2_ at specific corners of the gold nano-rectangles, ultimately yielding plasmonic structures with pronounced chirality. Subsequently, in 2019, Kim et al. investigated the formation of chiral superstructures from gold nanoparticles, facilitated by CPL (Figure 2b) [44]. The study underscored the photon-to-matter chirality transfer as a strategy for synthesizing chiral nanostructures. Despite the fleeting lifetime of plasmonic states, which has posed a challenge for the use of plasmonic nanoparticles in light-driven synthesis, the assembly into chiral nanostructures was accomplished, showcasing the potential of gold nanoparticles to retain the chirality of incident photons, thereby offering a method to fabricate diverse chiral nanomaterials. Furthermore, another innovative approach to synthesizing chiral nanoparticles has been reported. This study successfully demonstrated the preparation of chiral nanoparticles through the oxidative etching reaction of silver under circularly polarized light irradiation [46]. The ability to control chirality via such photochemical processes opens up new possibilities for designing materials with unique optical properties and enhanced performance in various applications.

In 2022, Xu et al. employed CPL to irradiate non-chiral gold nanoprisms with the addition of chiral dipeptides, leading to the secondary growth of non-chiral gold nanoprisms into chiral nanostructures (Figure 2c) [25]. The distinct shapes observed in chiral nanostructures are due to the selective deposition of gold at transient hotspots and the localized reduction of gold ions Au(III) to metallic gold Au(0). Given that the electric field is intensely concentrated at the corners of the trigonal nanoprisms serving as seeds, the morphology of the emerging nanoparticles is heavily influenced by circularly polarized light. These chiral Au nanoparticles exhibited a g-factor of up to 0.4, approximately five times higher than that of chiral nanoparticles obtained solely through induction and regrowth using chiral dipeptides. More recently, in 2024, Saito et al. prepared colloidal chiral AuNR@Ag nanostructures using circularly polarized light, eliminating the necessity for chiral molecules (Figure 2d) [47]. The chiral nanostructures were successfully prepared by depositing silver onto the surface of achiral AuNRs dispersed in a solution, under the influence of CPL. A noteworthy discovery was the detection of CD signals that mirrored the chirality of the CPL employed, particularly evident with the use of anisotropic gold nanorods or gold nanotriangles. Notably, no discernible CD signal was detected with spherical gold nanoparticles, highlighting the importance of the morphological anisotropy of the gold nanoparticles in achieving the desired chiroptical properties. Additionally, the research demonstrated the capability to fine-tune the chiroptical characteristics of the nanostructures by adjusting the wavelength of the CPL, thus presenting a viable strategy for crafting state-of-the-art materials with bespoke optical characteristics, suitable for a spectrum of applications from chiral detection to enantioselective catalytic processes.

## 3. Applications of Chiral Au Nanostructures

In recent years, chiral inorganic nanostructures have garnered considerable interest for their potential uses in biocatalysis, biosensing, and bioassays. While numerous studies have explored the applications of these chiral inorganic nanostructures, there is a scarcity of comprehensive reviews concentrating specifically on the applications of chiral gold nanomaterials. The unique ability of these nanomaterials to interact with biological systems based on their chirality has opened new avenues for therapeutic interventions against complex diseases like cancer and bacterial infections. In this review, we aim to discuss the recent progress of how chiral Au nanostructures can be applied to anti-bacterial, cancer therapy, biosensing, neuronal differentiation, and catalysis.

### 3.1. Chiral Gold Nanomaterials in Biomedical Applications

#### 3.1.1. Chiral Gold Nanomaterials in Antibacterial Applications

**Figure 3:**
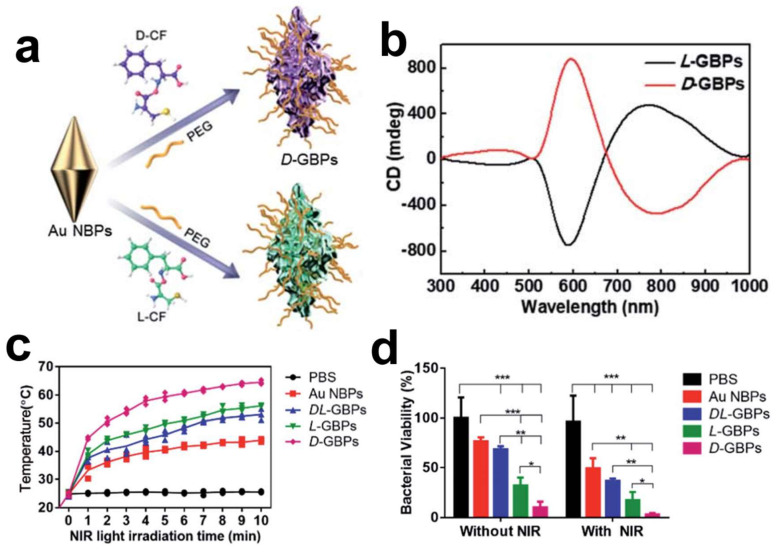
(**a**) Schematic diagram of D-/L-GBPs preparation. (**b**) CD spectra of D-/L-GBPs. (**c**) Temperature changes of *Staphylococcus aureus* solution under 808 nm laser irradiation. (**d**) Bacterial viability of *Staphylococcus aureus* following treatment with various techniques. * *p* < 0.05, ** *p* < 0.01, *** *p* < 0.001.

The escalating prevalence of antibiotic-resistant bacteria has rendered traditional antibiotics less effective, urgently calling for innovative therapeutic strategies in the fight against bacterial infections. Recent strides in nanotechnology, especially those involving chiral gold nanomaterials, have paved new paths for antibacterial applications. By utilizing gold bipyramids as seeds and D-/L-Cys-Phe (CF) dipeptides as chiral ligands, chiral gold nano-bipyramids (GBPs) with a unique sea cucumber-like shape have been crafted, exhibiting an optical anisotropy factor of 0.102 at a wavelength of 573 nm. The chiral GBPs displayed robust antibacterial activity both in vitro and in vivo against formidable pathogens, such as *Staphylococcus aureus*. Notably, these chiral GBPs, including D-GBPs and L-GBPs, demonstrated significant antibacterial efficacy, with in vitro bacterial mortality rates reaching 98% and 70%, respectively. Mechanistic inquiries revealed that the superior antibacterial action of D-GBPs is significantly attributed to their high binding affinity towards protein A of *Staphylococcus aureus* (Figure 3) [48]. Furthermore, the peptide-directed synthesis of these chiral nano-bipyramids holds promise in efficiently eradicating bacteria and facilitating wound healing in vivo. The study underscores the significance of the nanoparticles’ chiral properties in binding with bacterial proteins, leading to the physical disruption of bacterial cell walls and thereby enhancing therapeutic efficacy against bacterial infections [48]. The antibacterial mechanisms exhibited by chiral GBPs are multifaceted. Chiral GBPs with sharp, spike-like structures exhibit a high binding affinity to bacterial proteins, resulting in the effective destruction of bacterial cell walls, while their photothermal properties, activated under near-infrared light, further bolster their antibacterial capabilities. Notably, D-GBPs have demonstrated superior performance in promoting wound healing and preventing sepsis in mouse models, underscoring their capacity as a prospective treatment for combating bacterial infections.

#### 3.1.2. Chiral Gold Nanomaterials in Cancer Therapy

Cancer, ranked as the globe’s second most common cause of mortality, poses a formidable challenge to public health. Traditional clinical strategies for combating cancer encompass surgery, chemotherapy, and radiation therapy. However, the efficacy of these standard treatments is often undermined by issues such as tumor recurrence, inadequate tumor targeting, and resistance to radiation. In this context, chiral gold nanoparticles have risen to prominence as a potential cancer treatment modality, attributed to their capacity to selectively and efficiently trigger autophagy and apoptosis in cancer cells. Nie’s group investigated the impact of chiral gold nanoparticles (D-Au and L-Au) on cellular metabolic reprogramming, particularly focusing on macrophage activation. D-Au particles showed a superior ability to activate macrophages compared to their L-Au counterparts. These findings underscore the importance of understanding how chiral materials influence metabolic pathways in immune cells, which is crucial for developing more effective chiral-based anticancer therapies [49].

**Figure 4:**
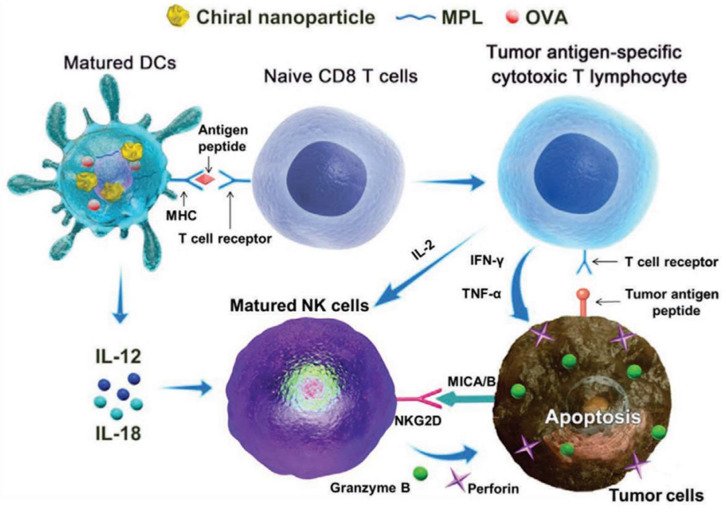
Schematic illustration of chiral AuNP-based cancer immunotherapy mechanism [50].

Xu’s group first discovered that chiral gold nanomaterials can induce differential immunological effects in mice [25], and subsequently, the team further confirmed that chiral gold nanomaterials, when used as adjuvants, can exert therapeutic and preventive effects in tumor immunology. As shown in Figure 4, chiral gold nanoparticles (NPs) showed remarkable efficacy in targeting NK cells and CD8+ T cells for cancer immunotherapy [50]. Chiral AuNPs were shown to significantly enhance both innate and acquired immunity against tumor growth, surpassing the effects observed with their achiral counterparts. Notably, L-type NPs induced a considerably higher proportion of CD8+ T and CD69+ NK cells compared to D-type NPs, underscoring the impact of nanoparticle chirality on immunological outcomes. These findings underscore the potential of chiral gold NPs to serve as potent immuno-adjuvants, paving the way for new cancer treatment strategies that harness the body’s immune system to combat tumors more effectively.

**Figure 5:**
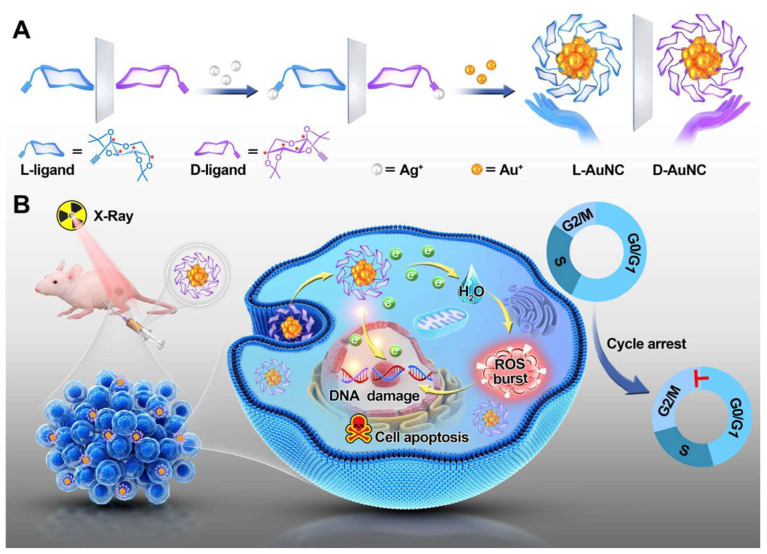
Schematic illustration of (**A**) the synthesis of L-/D-AuNC and (**B**) their application in chiral gold nanocluster-based cancer radiotherapy via ROS burst, cell cycle arrest and DNA damage [51].

Additionally, chiral gold nanoclusters have been employed in the radiotherapy of tumors [51]. Zhao’s group synthesized a pair of enantiomeric alkyl-protected L-/D-gold nanoclusters (termed L-/D-AuNCs), which exhibit strong CD and circularly polarized luminescence activity, enhancing the chirality-dependent effect in tumor radiotherapy (Figure 5). The D-type AuNCs demonstrated superior radiosensitizing effects in vitro compared to the L-type AuNCs. The D-type AuNCs have lower cytotoxicity, better dispersibility in biological systems, and higher radiosensitizing efficiency than the L-type AuNCs. In the animal model experiments for tumor therapy, the administration of D-type AuNCs alongside radiation therapy led to a roughly 51% decrease in tumor size in mice, contrasting with those treated solely with X-ray irradiation. Notably, there were no signs of pathological damage to the major organs observed. This research underscores the significance of chirality in the field of radiotherapy and paves the way for the development of innovative radiosensitizing agents. Furthermore, Spaeth et al. provided valuable insights into the use of chiral gold nanorods for photothermal therapy [52].

#### 3.1.3. Chiral Gold Nanomaterials in Cell Differentiation

**Figure 6:**
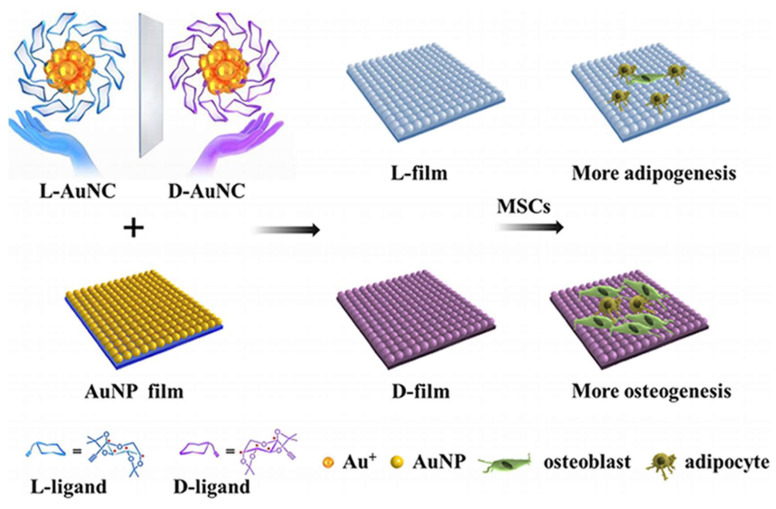
Schematic illustration of MSCs grown and differentiated on L/D-Au films [53].

Chiral gold nanomaterials hold substantial potential in tissue regeneration and cell differentiation. Kang et al. investigated the impact of chiral L-/D-AuNCs loaded on a two-dimensional gold nanoparticle film (L-/D-film) on the adhesion and differentiation of mesenchymal stem cells (MSCs) (Figure 6) [53]. The D-film exhibited superior biocompatibility compared to the L-film, likely attributed to distinct adsorption behaviors of serum proteins on chiral surfaces. Under adipogenic/osteogenic induction, the D-film demonstrated greater osteogenic differentiation than the L-film, which led to increased adipogenic differentiation. The expression levels of RUNX2 also confirmed the diversity in differentiation. These findings indicate that chirality at the nanocluster scale can significantly influence cell behavior, aiding in understanding the selection of chirality in biological systems across different scales and providing insights for the development of the next generation of chiral extracellular matrices.

Zhang et al. explored the effects of chiral cysteine-modified AuNPs (L-/D-Cys-AuNPs) on cell differentiation [54]. This investigation delves into the efficacy of L-/D-Cys-AuNPs in the realm of tissue regeneration, with a particular emphasis on their influence on osteogenic differentiation and autophagy within human periodontal ligament cells (hPDLCs) and the subsequent regeneration of periodontal tissue. The study discerned that L-Cys-AuNPs were more readily internalized by hPDLCs than their D-Cys counterparts, markedly enhancing the expression of pivotal osteogenic indicators and proteins associated with autophagy in vitro. Utilizing a rat model with periodontal defects, it was observed that L-Cys-AuNPs were more proficient in fostering the regeneration of periodontal tissue. The research posits that the induction of autophagy in L-Cys-AuNP-treated hPDLCs could be pivotal for cellular differentiation and tissue regeneration. Collectively, L-Cys-AuNPs demonstrated superior performance over D-Cys-AuNPs in terms of cellular uptake, the regulation of autophagy, osteogenic differentiation, and the regeneration of periodontal tissue. This underscores the promise of L-Cys-AuNPs in periodontal regeneration and sheds new light on the utilization of chirality-modified bioactive nanomaterials. The study also preliminarily elucidates the substantial role of chirality in modulating stem cell differentiation and periodontal tissue regeneration, uncovering the potential applications and underlying mechanisms of L-Cys-AuNPs in this context, thus providing a novel lens through which to examine chirality-modified bioactive nanomaterials.

#### 3.1.4. Chiral Gold Nanomaterials in Neurodegenerative Disease Therapy

**Figure 7:**
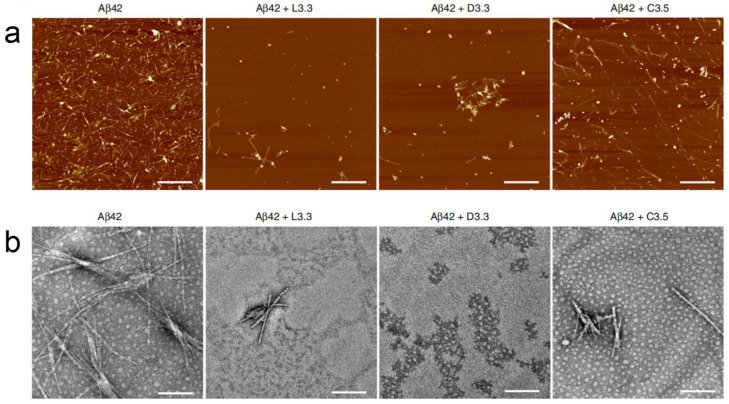
(**a**) AFM images of Aβ42 (40 μM) in the absence and presence of L3.3, D3.3, or C3.5 (110 nM) after co-incubation for 48 h. Scale bars, 1 μm. (**b**) TEM images of Aβ42 (40 μM) in the absence and presence of L3.3, D3.3, or C3.5 (110 nM) after co-incubation for 48 h. Scale bars, 200 nm [56].

Neurodegenerative diseases encompass a group of progressive neurological conditions marked by ongoing, degenerative alterations and the degeneration of neurons across different brain areas, with Alzheimer’s disease (AD) and Parkinson’s disease (PD) being the most prominent examples. Due to their multi-system involvement, these diseases pose significant therapeutic challenges. Chiral gold nanomaterials have been applied to the treatment of AD. Tang’s group reported that both 3.3 nm L- and D-glutathione-stabilized gold nanoparticles (denoted as L3.3 and D3.3, respectively) are capable of inhibiting Aβ42 aggregation and can cross the blood-brain barrier (BBB) following intravenous administration without noticeable toxicity. Further investigation revealed that, compared to L3.3, D3.3 possesses a greater binding affinity for the chiral molecule Aβ42 and achieves higher brain biodistribution, resulting in more effective inhibition of Aβ42 fibrillation and better amelioration of behavioral impairments in AD model mice.(Figure 7).

In 2023, Xu’s group employed uniquely chiral-shaped gold nanoparticles to intervene in the gut microbiota of mice; left-handed AuNPs significantly increased the abundance of *Lactobacillus* and *Clostridium*, while significantly decreasing the abundance of *Enterobacteriaceae* and *Desulfovibrio*. This not only protected the integrity of the intestinal mucosal barrier but also doubled the levels of indole-3-acetic acid in the blood, improving the brain’s immune environment. The promotion of tryptophan metabolism to indole-3-acetic acid led to a notable reduction in amyloid-beta (Aβ) and phosphorylated tau protein (p-τau) in the brains of AD mice, thereby restoring their cognitive abilities [55].

**Figure 8:**
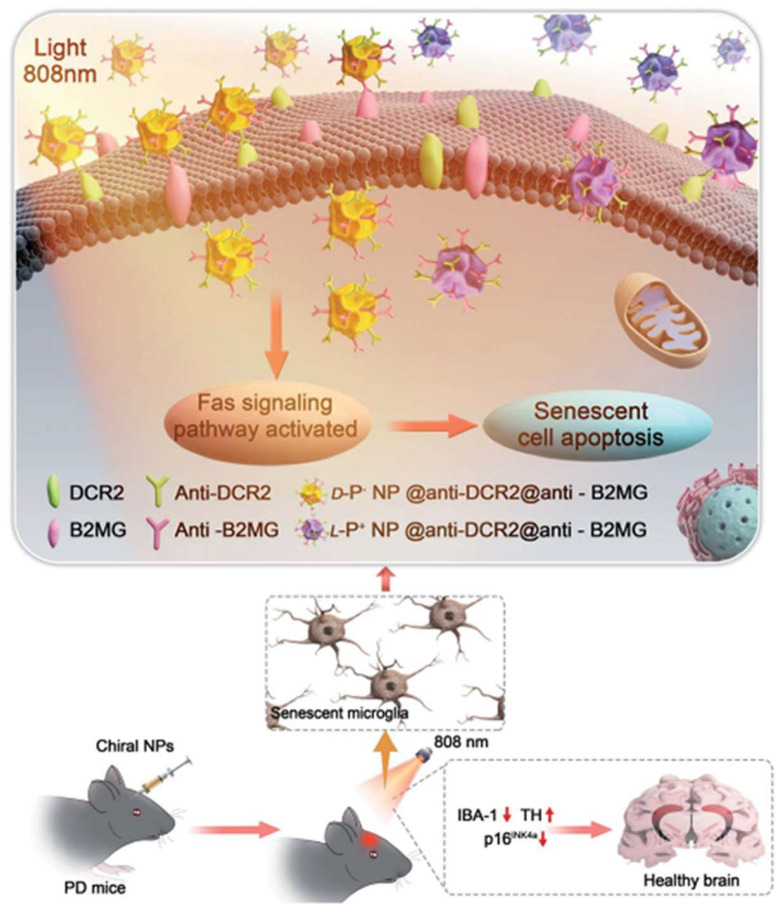
Scheme of the apoptotic pathways triggered in aged microglia cells by chiral gold nanoparticles upon exposure to an 808 nm laser in the brain of mice with Parkinson’s disease [57].

Furthermore, chiral gold nanomaterials have also been applied in the treatment of PD. Xu et al. showed that chiral Au nanoparticles can selectively eliminate senescent microglia in a mouse model of PD, thereby ameliorating symptoms associated with age-related neurodegenerative conditions (Figure 8) [57]. Specifically, L-AuNPs exhibited higher clearance efficiency of senescent cells in vitro compared to D-AuNPs, without harming normal cells. Mechanistic studies revealed that chiral NPs mediated the apoptosis of senescent cells through the activation of the Fas signaling pathway. In PD mouse models, L-AuNPs effectively cleared senescent microglia in the brain upon light irradiation, reducing the levels of α-synuclein in the cerebrospinal fluid. Additionally, mice treated with L-AuNPs showed significant improvements in motor function and spatial memory tests, indicating the potential therapeutic efficacy of this treatment strategy for PD.

### 3.2. Chiral Gold Nanomaterials in Biosesing

The identification and measurement of chiral molecules play a crucial role in fields such as analytical chemistry, chemical biology, pharmaceuticals, and agrochemicals. Currently, the common methods for chiral detection are primarily divided into two categories: chromatography and spectroscopy. Chromatography mainly utilizes the selective adsorption capacity of chiral molecules by chromatographic columns for separation, purification, and analysis. However, for different chiral molecules, chromatography requires the selection of different columns and mobile phases, leading to its limited universality, high operational difficulty, and high analytical costs. Moreover, spectroscopy cannot be used for racemic mixtures and enantiomers with weak optical interactions. Chiral gold nanomaterials offer new opportunities for identifying and quantifying chiral compounds [58,59,60].

#### 3.2.1. Chiral Gold Nanomaterial-Based SERS Sensors

**Figure 9:**
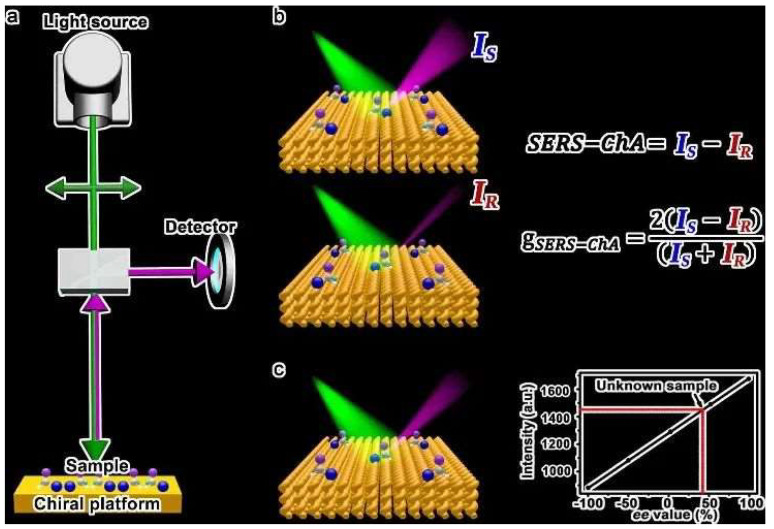
Schematic depiction of the SERS-ChA technique for distinguishing enantiomers. (**a**) Conventional Raman scattering equipment with a chiral platform. (**b**) SERS-ChA and g-factor are obtained for enantiomers. (**c**) Absolute configurations and ee (enantiomeric excess) values of unknown samples can be obtained from the calibration curves [68]. I_S_ represents the SERS intensity corresponding to the signal from the S-enantiomer, while I_R_ represents the SERS intensity from the R-enantiomer.

Over recent years, substantial progress has been made in the engineering of Raman sensors utilizing chiral gold nanomaterials [61,62,63,64,65,66,67]. In 2020, Che’s group introduced chiral nanostructured gold films (CNAFs) as a SERS substrate to create Surface-Enhanced Raman Scattering Chiral Anisotropy (SERS-ChA), which distinguishes enantiomers through SERS with incident linearly polarized light (Figure 9) [68]. SERS-ChA is the selective enhancement of the Raman scattering of enantiomer molecules on chiral plasmonic metal surfaces, exhibiting high enantiomeric sensitivity and overcoming the shortcomings of optical rotation systems and chromatography. This method is universal, requiring no sample separation or purification, regardless of molecular size, polarity, or chromophore presence; it can qualitatively and quantitatively detect all enantiomer molecules using a single substrate.

The misfolding and clumping of proteins are implicated in a range of illnesses, including AD, PD, Huntington’s chorea, and other conditions characterized by neurodegeneration. Consequently, a thorough comprehension of protein architecture is essential for grasping and managing these neurodegenerative disorders. The abnormal aggregation of beta-amyloid (Aβ) protein results in the formation of amyloid fibrils, which are intricately linked to the onset and progression of neurodegenerative diseases such as AD. This makes the ultra-sensitive detection of Aβ protein crucial for the early diagnosis of AD. Wang and colleagues utilized hollow platinum (Pt) nano-triangular rings as seeds and L/D-glutathione (L/D-GSH) as chiral ligands to induce the orderly growth of chloroauric acid on hollow Pt triangular rings, fabricating chiral triangular nano-gold rings with a Pt skeleton (L/D-Pt@Au triangular nano-rings, referred to as L/D-Pt@Au TNRs) [61]. The L/D-Pt@Au TNRs exhibited strong chiroptical activity, with a chiroptical anisotropy factor (g-factor) value reaching 0.023. Due to the resonant coupling between the plasmon of the chiral triangular nano-rings and the induced electric and magnetic dipoles of molecular enantiomers, the SERS intensity of chiral enantiomers is differentially enhanced. Moreover, Pt@Au TNRs of different chiral configurations show varying affinities for different folding and aggregation states of Aβ protein, enabling the chiral triangular nano-rings to effectively discriminate enantiomeric types and quantitatively analyze the structures of Aβ monomers and fibrils. For Aβ42 monomers and fibrils, the LOD can be as low as 45 fM and 4 fM, respectively. Additionally, the chiral D-Pt@Au TNR can also serve as a biosensor for the quantitative sensing of Aβ42 in the cerebrospinal fluid of AD patients, with the sensor capable of identifying picogram levels of Aβ42 with ultra-high sensitivity. This work provides a novel synthesis method for hollow chiral plasmonic nanostructures and serves as a biosensor that will play a key role in the study of beta-amyloid plaques in AD, PD, and other neurodegenerative diseases, as well as in clinical early diagnosis.

Leong et al. reported that by coupling asymmetric nano-porous gold bowls (NPGBs) with electrochemical SERS (EC-SERS), enantioselective molecular fingerprints were obtained, permitting immediate, label-free enantioselective SERS differentiation and the measurement of numerous biologically significant chiral molecules [64]. The intrinsic enantioselectivity of NPGB composites, where surface atomic defects on the NPGBs serve as localized asymmetric sites, prompt stereospecific analyte interactions with the NPGBs. Notably, this was accomplished without chiral molecules and with consistent batch-to-batch reproducibility. By combining EC-SERS with chiral NPGBs, the surface interactions of analytes can be manipulated using an external potential, enhancing both interaction and SERS signal amplification, thereby achieving superior enantioselectivity. Employing L- and D-tryptophan (TRP) as a model pair of enantiomers, in situ EC-SERS studies coupled with density functional theory (DFT) analyses disclosed enantioselective TRP–NPGB interactions, evidenced by the emergence of enantioselective SERS peaks and significant alterations in peak intensity ratios. This chiral discrimination is unique to NPGBs, with smooth Au nanoparticles showing no enantioselective spectral differences. Combining chemometric analysis, the accurate quantification of enantiomeric compositions was achieved, with a cross-validation R^2^ value of 0.96. Finally, to emphasize the versatility of their strategy, the enantiomeric fingerprint spectra of an additional pair of enantiomers were revealed, demonstrating the ability to detect a variety of enantiomers. This research pioneers label-free chiral SERS detection, marking significant progress in its practical applications across various fields.

**Figure 10:**
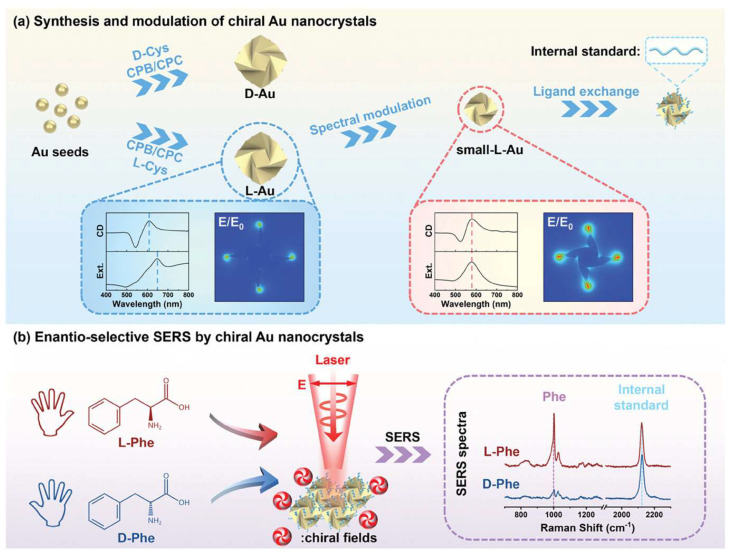
Schematic illustration of (**a**) synthesis and modulation of chiral Au nanocrystals, and (**b**) enantioselective SERS by chiral Au nanocrystals [62].

More recently, in 2024, Niu’s group presented the development of a reliable enantioselective SERS substrate using chiral gold nanocrystals with finely modulated chiral fields and internal standards [62] (Figure 10). The study demonstrates that by enhancing chiral electromagnetic fields and employing internal standards, the SERS stability is significantly improved, leading to a stable six times difference in SERS ratio for L- and D-phenylalanine. Theoretical simulations reveal that linearly polarized light can excite the chiral fields of chiral Au nanocrystals, converting non-chiral far-field light into chiral near-field sources. The mechanism of enantioselective SERS is elucidated by the scattering difference of chiral molecules in chiral near fields. This work paves the way for the development of enantioselective SERS and related chiroptical technologies, offering a robust SERS substrate for discriminating molecular chirality and a design concept for a broad range of chiral sensing applications.

#### 3.2.2. Chiral Gold Nanomaterial-Based Electrochemical Sensors

Utilizing the excellent electrochemical properties and chiral recognition capabilities of chiral gold nanoparticles, chiral electrochemical sensors can be developed for the selective detection of enantiomers. In 2022, Niu’s group disclosed a scalable wet chemical synthesis approach to generate high-Miller-index facets in L- and D-Au nanocrystals, respectively [69]. The study probed into the growth mechanisms of these homochiral facets, uncovering a novel pathway for nanocrystal development. Notably, the research highlighted the exceptional enantioselective recognition capabilities of these homochiral surfaces, facilitating an efficient electrochemical technique for distinguishing L-/D-tryptophan. This contribution establishes a basis for in-depth studies on heterogeneous enantioselective reactions and could lead to the creation of nanocatalysts tailored for enantioselective chemistry, with a specific emphasis on their analytical detection potential.

#### 3.2.3. Other Chiral Sensors Based on Chiral Gold Nanomaterials

In addition to the aforementioned chiral electrochemical sensors and chiral SERS sensors, chiral gold nanomaterials can also be employed to develop other types of sensors. Cai et al. utilized gold nanoparticles to self-assemble into chiral gold nanofilms, enabling precise differentiation and recognition of circularly polarized light [70]. They established a relationship between photocurrent and light polarization degree, allowing for highly sensitive detection of the polarization of incident light beams. Importantly, the detection of CPL is unaffected by the angle of light incidence, demonstrating consistent photocurrent results across a range of incidence angles from 45° to 90°. This characteristic of the chiral gold nanofilms, which is independent of the angle of light incidence, significantly enhances the accuracy of CPL detection.

In 2023, Lu and colleagues devised an uncomplicated, economical, and adaptable method for fabricating a range of chiral metamaterials that are highly regular and customizable [71]. The method involves the use of polarization-directed growth, which leverages the self-aligned near-field to enhance photochemical growth along the polarization direction, resulting in oriented nanostructures. The obtained plasmonic chiral Au nanostructures showed potential for sensing different chiral enantiomers. The Au chiral nanostructures with left-handed (LH) and right-handed (RH) rotations show different responses to LCP and RCP light, leading to opposite responses in circular differential scattering (CDS). The handedness of the plasmonic spiral configurations can be adjusted by altering the alignment angle, with the peak CDS detected at a 45° orientation. The binding of chiral molecules to these structures induces varying degrees of plasmon resonance shifts, quantifiable through the g-factor. For chiral molecules like L-cysteine, the detection threshold is as low as 0.01 mg/mL. Provided that the chiral plasmonic chip remains uncontaminated, it can be reused for the analysis of additional chiral species. The research indicates that L-enantiomers consistently show negative dissymmetric factors, which aids in chirality discrimination. Conversely, for achiral molecules such as 1,2-ethanediol, the dissymmetric factor is null, confirming the accuracy of the detection method. Conversely, for non-chiral molecules like 1,2-ethanediol, the dissymmetric factor is zero, validating the measurement method.

In 2022, Kim et al. developed a chiral sensor that leverages the collective resonances (CRs) of assembled 2D crystals composed of isotropic, 432-symmetric chiral gold nanoparticles, known as helicoids [13]. The essence of this enantioselective sensing strategy is the interaction between CRs and chiral molecules. The energy redistribution resulting from the molecular back action on the chiral near-field propels the CRs in directions that are opposite to each other, contingent upon the chirality of the analyte. This optimizes the modulation of the collective CD, leading to an exceedingly sensitive and robust detection mechanism resilient to random molecular fluctuations. Gold helicoids 180 nm in diameter demonstrated the highest collective CD, with a tenfold increase in CD intensities at the extremities compared to other helicoids. This refined generation of CRs and collective CD could amplify the CD intensity by 2.5 times that of a randomly distributed helicoid suspension. The platform’s versatility as an enantioselective biomolecular sensor was further showcased by detecting microRNA-21 (a biomarker for lung cancer) with a limit of detection (LOD) of 114 pM. The method offers reliable and high sensitivity for detecting chiral molecules and has the potential for the in situ monitoring of conformational changes at molecular resolution.

In addition to the detection of enantiomers, chiral gold nanomaterials can also be utilized for enantiomeric separation. Liu et al. reported that L-Cys-modified Au nanoparticles could serve as a scaffold and spacer material in the fabrication of multifunctional separation membranes from graphene oxide nanosheets [72]. These separation membranes employ gold nanoparticles as robust spacers, preventing the collapse of graphene oxide sheets, and thereby ensuring stability. Moreover, the amphiphilic nature of L-Cys allows for the regulation of the interlayer spacing of graphene oxide sheets between 16.9 and 17.8 Å through pH adjustment, enabling controllable particle size sieving and ultra-high permeability. Ultimately, the membrane exhibits high enantioselectivity towards penicillamine (Pen) racemic mixtures, with a chiral separation factor and permeation rate reaching 1.83 and 21.7 L m^−2^ h^−1^ bar^−1^, respectively.

### 3.3. Chiral Gold Nanomaterials in Catalysis

**Figure 11:**
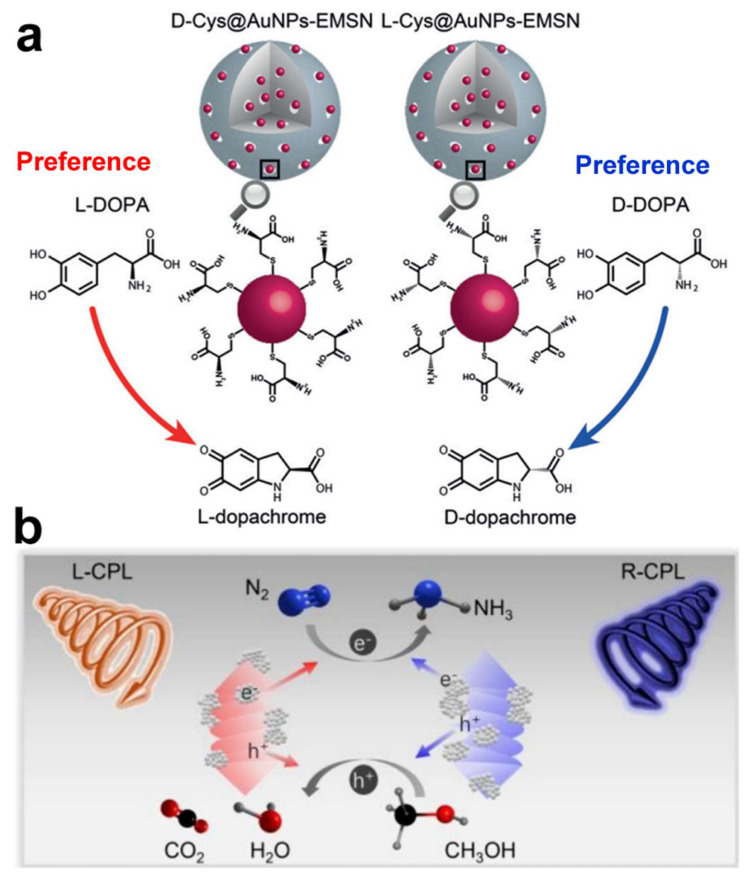
(**a**) Schematic illustration of enantioselective oxidation of chiral DOPA by d-Cys/l-Cys@AuNPs-EMSN [74]. (**b**) Schematic illustration of nitrogen fixation by chiral L/D-Au@CeO_2_ helical nanorods (HNRs) under CPL illumination [75].

Chiral gold nanomaterials hold broad application prospects in the field of catalysis, with their chiral properties giving them unique advantages in asymmetric catalysis and biocatalysis [40,73,74,75,76,77]. By meticulous surface modification, chiral gold nanomaterials can mimic the chiral catalytic characteristics of natural enzymes, thereby achieving enantioselective catalytic reactions. A chiral nanozyme (D-/L-Cys@AuNPs-EMSN) was synthesized, using AuNPs as the active center, chiral cysteine as the stereoselective recognition ligand, and extended mesoporous silica (EMSN) as the scaffold. The catalytic performance of the nanozyme was tested using the chiral substrate 3,4-dihydroxyphenylalanine (DOPA). The D-Cys@AuNPs-EMSN showed higher specificity for L-DOPA, while the L-Cys@AuNPs-EMSN showed higher specificity for D-DOPA (Figure 11a) [74]. By determining kinetic parameters and activation energy, coupled with molecular dynamics (MD) simulations, it was deduced that the establishment of hydrogen bonds between chiral Cys and DOPA is the primary source of chiral selectivity. This research introduces a novel class of chiral nanozymes capable of emulating the stereoselective catalytic activity of natural enzymes, holding promise for applications in pharmaceutical development and pesticide manufacturing.

In 2024, Li’s group employed a facile wet chemical method to selectively grow CeO_2_ on the surface of Au helical nanorods (HNRs), fabricating L/D-Au@CeO_2_ HNRs for the photocatalytic reduction of nitrogen gas [75]. The chiral Au@CeO_2_ HNRs, featuring a spatially separated structure of Au and CeO_2_, exhibited the highest photocatalytic performance in N_2_ fixation, which is 50.80 ± 2.64 times that of Au HNRs (Figure 11b). Furthermore, when L-Au@CeO_2_ HNRs were exposed to left circularly polarized light (CPL) and D-Au@CeO_2_ HNRs to right CPL, their photocatalytic efficiency was enhanced by 3.06 ± 0.06 times compared to samples irradiated with the opposite CPL, attributed to the asymmetric generation of hot carriers upon CPL excitation. This study not only provides a simple method to enhance the photocatalytic performance of chiral Au nanostructures but also demonstrates the application potential of chiral plasmonic materials in specific photocatalytic reactions, such as N_2_ fixation.

## 4. Conclusions and Outlook

This review article summarized the synthetic strategies of chiral gold nanomaterials and their application progress in various fields such as biomedicine, catalysis, and sensing. Chiral gold nanomaterials represent a groundbreaking class of nanomedicine, offering new possibilities for cancer immunotherapy and antimicrobial treatment. The unique chirality-dependent interactions between these nanoparticles and biological systems have demonstrated significant potential in enhancing immune responses and combating disease mechanisms at the molecular level. As research in this field advances, chiral gold nanomaterials are poised to revolutionize treatment strategies for a range of complex diseases, highlighting the crucial role of nanotechnology in advancing healthcare and medicine. Furthermore, interdisciplinary research has expanded the understanding of chiral gold nanomaterials, bridging concepts from physics, chemistry, and biology. This has led to deeper insights into the mechanisms controlling chiral interactions and their impact on material properties. As researchers continue to uncover the nuances of chirality in gold nanomaterials, the potential for new applications grows exponentially. The exploration of chiral gold nanomaterials marks a promising direction for the development of novel therapeutic agents. Future research should focus on optimizing the synthesis and functionalization of these nanoparticles to enhance their specificity and efficacy in targeting malignant cells and drug-resistant bacterial strains. Additionally, investigating the application of chiral gold nanomaterials in other diseases characterized by immune dysregulation may provide new therapeutic avenues.

Despite the numerous achievements in the synthesis and application of chiral gold nanomaterials, there are still many opportunities and challenges. Firstly, in terms of synthesis, there is a lack of simple, high-yielding methods for the production of gold nanomaterials with well-defined chiral configurations and strong chiral activity. Secondly, in applications, the CD signals of most chiral gold nanomaterials appear in the visible light region, which has a limited penetration depth. Therefore, to achieve a widespread application of chiral gold nanomaterials in biomedical photonics, the synthesis of gold nanoparticles with strong chiral activity in the near-infrared region is crucial. Thirdly, biosafety is a key factor for clinical application, and comprehensive research on the biosafety assessment of chiral gold nanomaterials is needed. Laboratory-based in vitro study models are inherently incapable of perfectly mimicking the in vivo conditions, which are necessary for precisely forecasting the biological interactions that occur within the human body. Other than considering the toxicity of nanomaterials, assessing the impact of size, shape, solubility, composition, crystallinity, and chirality-induced differential biological effects, factors such as cellular clearance rates and long-term in vivo stability also require more theoretical and experimental results for optimization. Future efforts should focus on fabricating surface-functionalized chiral gold nanomaterials endowed with various biomimetic properties, such as non-toxicity, stability in culture media, and cell/tissue specificity. As the transition from successful laboratory experiments to clinical practice continues, a deeper understanding of cellular uptake mechanisms of chiral gold nanoparticles, prediction of their biological impacts, and development of clinically valuable chiral gold nanoparticles is necessary.

In summary, the synthesis and application of chiral gold nanomaterials remain an extremely challenging yet fascinating research direction. We must continue to strive to improve the performance of chiral gold nanomaterials and expand their applications in various fields. It is believed that chiral gold nanomaterials will play a significant role in biomedicine, photonics, catalysis, and other areas in the future.

## Data Availability

Not applicable.
